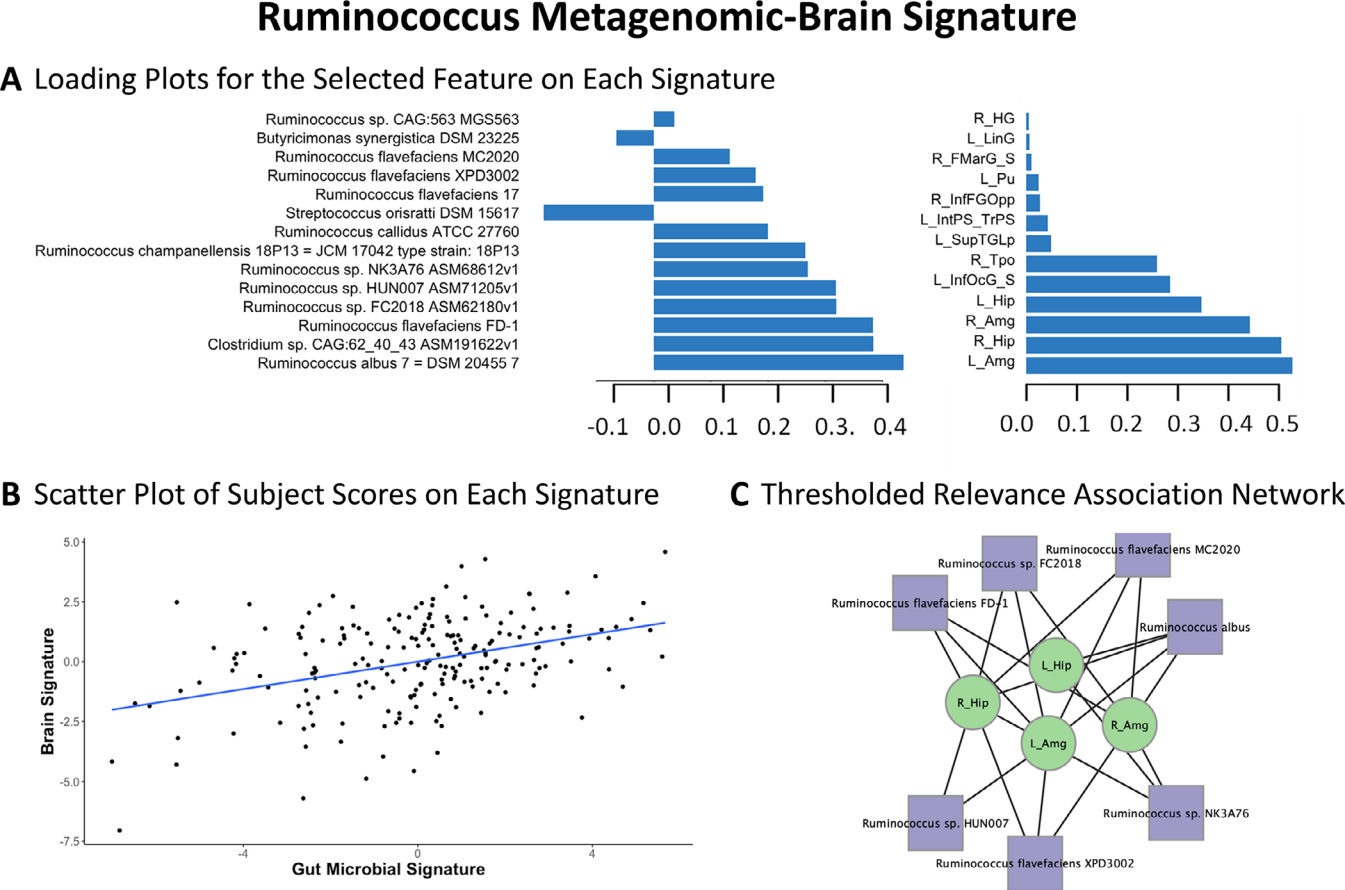# Ruminococcus‐based metagenomic‐brain signature linked to cognitive performance and Alzheimer’s disease markers

**DOI:** 10.1002/alz.089941

**Published:** 2025-01-09

**Authors:** Jennifer S Labus, Margo B. Heston, Emeran A Mayer, Ipsita Mohanty, Thai Tran, Kevin Huynh, Arpana Gupta, Federico E. Rey, Antonio González, Amanda H. Dilmore, Rob Knight, Rima Kaddurah‐Daouk, Barbara B. Bendlin, Alzheimer's Gut Microbiome Project Consortium (AGMP)

**Affiliations:** ^1^ Goodman‐Luskin Microbiome Center, University of California ‐ Los Angeles, Los Angeles, CA USA; ^2^ Oppenheimer Center for the Neurobiology of Stress and Resilience, David Geffen School of Medicine, University of California Los Angeles, Los Angeles, CA, Los Angeles, CA USA; ^3^ UCLA Vatche and Tamar Manoukian Division of Digestive Diseases, Los Angeles, CA USA; ^4^ Wisconsin Alzheimer's Disease Research Center, University of Wisconsin School of Medicine and Public Health, Madison, WI USA; ^5^ University of Wisconsin School of Medicine and Public Health, Wisconsin Alzheimer's Disease Research Center, Madison, WI USA; ^6^ Goodman Luskin Microbiome Center, Los Angeles, CA USA; ^7^ Skaggs School of Pharmacy and Pharmaaceutical Science, University of California‐San Diego, San Diego, CA USA; ^8^ UCLA Center for Neurobiology of Stress and Resilience, Los Angeles, CA USA; ^9^ University of Melbourne, Melbourne, VIC Australia; ^10^ Baker Heart and Diabetes Institute, Melbourne, VIC Australia; ^11^ La Trobe University, Melbourne, VIC Australia; ^12^ Department of Bacteriology, University of Wisconsin‐Madison, Madison, WI USA; ^13^ University of San Diego, La Jolla, CA USA; ^14^ University of California, San Diego, La Jolla, CA USA; ^15^ Center for Microbiome Innovation, University of California San Diego, La Jolla, CA USA; ^16^ Department of Medicine, Duke University, Durham, NC USA; ^17^ Duke University Medical Center, Durham, NC USA; ^18^ Department of Psychiatry and Behavioral Sciences, Duke University, Durham, NC USA; ^19^ Division of Geriatrics and Gerontology, Department of Medicine, University of Wisconsin School of Medicine and Public Health, Madison, WI USA; ^20^ Duke University, Durham, NC USA

## Abstract

**Background:**

The aim of this study was to identify a gut microbial signature associated with patterns of gray matter volume in AD, and to validate the microbial signature by testing it against measures of AD pathology and cognitive performance. Prior literature suggests that microbial species involved in bile acid production and inflammation may be implicated in the microbial signature.

**Method:**

The sample comprised 204 Microbiome in Alzheimer’s Risk Study participants (22 AD, 10 MCI, and 172 CN; 129 Females, 78 APOE+) from the Wisconsin Alzheimer’s Disease Research Center and Wisconsin Registry for Alzheimer’s Prevention. With shotgun metagenomic sequencing data and regional gray matter volumes calculated using T1‐weighted neuroimaging (Destrieux, Harvard Oxford subcortical atlases), sparse partial least squares regression was used to determine correlated sets of microbial features and gray matter regions that maximally explained the variance in the two datasets. General linear models were then used to link the derived microbial signature with an aggregated score of cognitive performance (PACC3), an estimated cognitive score (ADAS‐Cog‐13) derived using participants’ serum lipidomics, and amyloid and tau positivity status.

**Result:**

Greater abundance of 14 microbial features dominated by Ruminococcus species was associated (r=0.39, p=7.7e‐09) with greater volume in 13 regions dominated by the hippocampus, amygdala, and temporal regions, which are implicated in AD (Figure 1). This relationship was especially strong among APOE ε4 carriers. Higher scores on the microbial signature (associated with higher gray matter volume) were also associated with pTau negative status, and with better performance on the PACC3 and estimated ADAS‐Cog‐13.

**Conclusion:**

With roles in producing secondary bile acids and short chain fatty acids, Ruminococcus species may benefit the gut mucosal barrier and abrogate inflammatory mechanisms. This study suggests these microbes could protect against neurotoxic processes including tau hyperphosphorylation, resulting in preserved cortical volume and better cognitive performance.

**Funding**: Alzheimer Gut Microbiome Project, NIA U19 AG063744